# The accuracy of prediction of genomic selection in elite hybrid rye populations surpasses the accuracy of marker-assisted selection and is equally augmented by multiple field evaluation locations and test years

**DOI:** 10.1186/1471-2164-15-556

**Published:** 2014-07-04

**Authors:** Yu Wang, Michael Florian Mette, Thomas Miedaner, Marlen Gottwald, Peer Wilde, Jochen C Reif, Yusheng Zhao

**Affiliations:** Leibniz Institute of Plant Breeding and Crop Plant Research (IPK), Gatersleben, 06466 Germany; State Plant Breeding Institute, University of Hohenheim, Stuttgart, 70599 Germany; Syngenta Agro GmbH, Am Technologiepark 1-5, Maintal, 63477 Germany; KWS LOCHOW GMBH, Ferdinand-von-Lochow-Str. 5, 29303 Bergen, Germany

**Keywords:** Marker-assisted selection, Genomic selection, Cross-validation, Hybrid rye, Relatedness, Evaluation locations, Testing years

## Abstract

**Background:**

Marker-assisted selection (MAS) and genomic selection (GS) based on genome-wide marker data provide powerful tools to predict the genotypic value of selection material in plant breeding. However, case-to-case optimization of these approaches is required to achieve maximum accuracy of prediction with reasonable input.

**Results:**

Based on extended field evaluation data for grain yield, plant height, starch content and total pentosan content of elite hybrid rye derived from testcrosses involving two bi-parental populations that were genotyped with 1048 molecular markers, we compared the accuracy of prediction of MAS and GS in a cross-validation approach. MAS delivered generally lower and in addition potentially over-estimated accuracies of prediction than GS by ridge regression best linear unbiased prediction (RR-BLUP). The grade of relatedness of the plant material included in the estimation and test sets clearly affected the accuracy of prediction of GS. Within each of the two bi-parental populations, accuracies differed depending on the relatedness of the respective parental lines. Across populations, accuracy increased when both populations contributed to estimation and test set. In contrast, accuracy of prediction based on an estimation set from one population to a test set from the other population was low despite that the two bi-parental segregating populations under scrutiny shared one parental line. Limiting the number of locations or years in field testing reduced the accuracy of prediction of GS equally, supporting the view that to establish robust GS calibration models a sufficient number of test locations is of similar importance as extended testing for more than one year.

**Conclusions:**

In hybrid rye, genomic selection is superior to marker-assisted selection. However, it achieves high accuracies of prediction only for selection candidates closely related to the plant material evaluated in field trials, resulting in a rather pessimistic prognosis for distantly related material. Both, the numbers of evaluation locations and testing years in trials contribute equally to prediction accuracy.

**Electronic supplementary material:**

The online version of this article (doi:10.1186/1471-2164-15-556) contains supplementary material, which is available to authorized users.

## Background

Rye (*Secale cereale* L.) is an important European crop used for food, feed, and bioenergy that is grown primarily in Eastern, Central and Northern Europe. The main rye belt stretches from the northern parts of Germany through Poland, Ukraine, Belarus, Lithuania and Latvia into central and northern Russia. In contrast to the other major crops of the *Triticeae* tribe, barley (*Hordeum* sp.) and wheat (*Triticum* sp.), rye is an outbreeding species in which selfing is usually prevented by a gametophytic self-incompatibility system [1, 2]. As an alternative to open-pollinated varieties, hybrid breeding has been established based on a cytoplasmatic-genic male sterility (CMS) system [3]. Hybrid rye breeding started in 1970 at the University of Hohenheim in Germany and the first hybrid varieties were released in Germany in 1984 [4]. Economically important traits in hybrid rye are, among others, grain yield and plant height in context of productivity as well as starch content and total pentosan content with regard to end user quality [5].

Current plant breeding programs are making extensive use of molecular markers to predict the performance potential of the involved plant material. In particular, marker-assisted selection (MAS) is widely applied, but is not necessarily an optimal approach for complex agronomic traits as it is usually based on predictions derived from only a few markers in linkage disequilibrium (LD) to large effect quantitative trait loci (QTL) and, thus, ignores the contributions from small to intermediate effect QTL [6, 7]. To advance the accuracy of prediction, genomic selection (GS) has been suggested as an extension of MAS [8]. In GS, a large number of molecular markers distributed evenly over the genome is used to train the prediction model. Sufficient marker density provided, GS potentially makes use of all the genetic variance present in an analyzed population by summing the effects of all individual markers [7] and thus can be expected to also include information from small effect gene loci that cannot be captured by traditional QTL determination via MAS [9]. Accordingly, GS is of growing importance for efficient and cost-effective breeding programs [8].

In this context, cross-validation approaches have become an important tool for the empirical evaluation of the accuracy of prediction. Here, a population of plants for which phenotyping and high density genotyping data are available is split into two subsets, an estimation set and a test set. Marker effects are then determined based on the data from the estimation set, followed by the prediction of the genotypic values of the plants in the test set based on the estimated marker effects. The correlation of predicted and observed values in the test set provides a measure for the accuracy of prediction. This has been applied to e.g. test-cross populations of maize [10–13], wheat [14, 15] and barley [16, 17].

Several analytical approaches based on different assumptions with regard to the marker effects have been proposed for GS [8, 18]. Bayesian methods such as Bayes A estimate the variances of the effects of markers separately, while ridge regression best linear unbiased prediction (RR-BLUP) assumes that all marker effects are normally distributed and have identical variance [8]. RR-BLUP has been proved to afford high prediction accuracies across crops and traits [16] and is suitable for GS of complex traits [19, 20].

In two bi-parental segregating populations used in test-crosses to produce hybrid rye, grain yield, plant height, starch content, and total pentosan content were reported to represent quantitative traits controlled by multiple small to medium effect QTL [5]. Using data sets from this material, we show in our current study that GS has a consistently superior accuracy of prediction in comparison to MAS. Relatedness of the plant material included in the estimation and test sets clearly affects the accuracy of prediction, and limiting the number of locations in field testing has almost the same impact than limiting the number of years. This supports the view that establishing calibration models for GS requires phenotyping across locations and years.

## Methods

### Plant material and field experiments

The plant materials and field experiments used to obtain the data sets analyzed in this study are described in detail in Miedaner et al. [5]. In brief, three elite winter rye inbred lines (Lo90-N, Lo115-N and Lo117-N) were used as parents to generate segregating population A (Pop-A, Lo115-N × Lo90-N) and population B (Pop-B, Lo115-N × Lo117-N), respectively. F_1_ plants from crosses of parental lines were self-pollinated under isolation bags during two generations to obtain F_3_ plants by single seed descent. From each population, Pop-A and Pop-B, 220 randomly selected F_3_ progenies were used for pollination of a cytoplasmically male sterile (CMS) single cross tester (X × Y) between isolation walls resulting in three-way hybrids of the type (X ● Y) × F_3:4_. The CMS tester was genetically unrelated to the parents of both populations.

Field experiments with these hybrids were carried out in two years (2010 and 2011) at five locations, Wohlde (WOH, Germany, N52.8°, E10.0°, 80 m above sea level), Beckedorf (BEK, Germany, N52.5°, E10.3°, 80 m above sea level), Petkus (PET, Germany, N51.6°, E13.2°, 130 m above sea level), Stuttgart/Hohenheim (HOH, N48.4°, E9.1°, 400 m above sea level), and Walewice (WAL, Poland, N52.6°, E19.4°, 184 m above sea level). The respective location × year combinations are denoted as environments WOH10, WOH11, BEK10, BEK11, PET10, PET11, HOH10, HOH11, WAL10 and WAL11 throughout this study.

Hybrid collections derived from segregating F_3:4_ lines from both populations were evaluated together with hybrids obtained from their parental lines (repeated 9 times) as well as 2 common checks in field traits using an incomplete 24 × 10 alpha design with two replications. Data for grain yield (dt ha^-1^), plant height (cm), starch content (%) and total pentosan content (%) of hybrids were obtained as described by Miedaner et al. [5]. Starch content (%) and total pentosan content (%) were determined by near-infrared reflectance spectroscopy (NIRS) recorded with a Bruker MPA FT- NIRS instrument (Bruker Optics Ettligen) in reflectance mode over the range from 850 to 2500 nm. The samples were scanned twice in duplicate repacking using two different Petri dishes of 8.7 cm diameter as sampling cups on a rotating device with on average 32 scans in 10 seconds. Prediction models were calculated with OPUS Software version 6.5 (Bruker Optics Ettligen). Calculations were carried out with a modified partial least squares (PLS) procedure using a 1st derivation and a scatter correction (SNV) of the spectra. Samples were randomly assigned to calibration and validation sets, and calibration was performed based on chemical quantification methods [21]. Finally, the suitability of the models was controlled within the validation set. For grain yield and plant height, data across nine environments (BEK10, BEK11, PET10, PET11, HOH10, WAL10, WAL11, WOH10, and WOH11) were included in our analysis. Data from HOH11 were not used due to low repeatability (0.01 in Pop-A and 0.00 in Pop-B for grain yield and 0.69 in Pop-A and 0.19 in Pop-B for plant height, respectively). For starch content and total pentosan content, data across six environments (PET10, PET11, WAL10, WAL11, WOH10, and WOH11) were included.

### Phenotypic data analysis

Best linear unbiased estimates (BLUEs) for testcross progenies across environments were determined by the restricted maximum likelihood method using ASReml version 3.0 [22] based on a two-step linear regression model:

Step 1
1

where *y*_Env_ refers to the BLUEs of each plot, 1_n_ is a vector with the length n equal to the number of genotypes times the number of replications, *μ* denotes the overall mean, *G* is a design matrix for fixed effects of the genotypes, *α*_G_ refers to a N-vector of the genotype effects with N equal to the number of genotypes, *R* is a design matrix for random effects of the replication, *α*_R_ represents a vector of the replication effects, *B* is a design matrix for random effects of the block, *α*_B_ refers to a vector of the block effects and *e* is a residual term. With step 1, BLUEs of testcross progenies within each environment were estimated, which were then applied in step 2 to estimate BLUEs of testcross progenies across nine or six environments, respectively.

Step 2
2

where *y* refers to the BLUEs across all the environments, 1_k_ is a vector with the length k equal to the number of genotypes times the number of environments, *E* is a design matrix assigning random environment effects to the phenotypes, *α*_Env_ is a vector of environments effects, *F* denotes a design matrix of random interaction effects of genotype × environment, *α*_F_ is a vector of interaction effects and *e* is a residual term.

The same linear regression model, in which in both, step 1 and step 2, *G* can be viewed as a design matrix for random effects of the genotypes, was applied to estimate the variance components, including genotypic variance ( ), genotype × environment interaction variance ( ) and variance of effective error ( ) across both segregating populations (Table 1). Heritability (*h*^2^) was estimated as , where  is the genotypic variance across nine (for grain yield and plant height) or six (for starch content and total pentosan content) environments, respectively. *Nr.Env.* and *Nr.Rep.* refer to the number of environments and replications, separately, and  denotes the variance of effective error across nine or six environments, respectively [23]. The broad-sense heritability of each environment, denoted as repeatability (*r*), was calculated as , where  and  are the genotypic variance and the variance of effective error within each environment, respectively [23].

**Table 1:** **Estimates of variance components and heritability** (***h*** ^***2***^) **for grain yield, plant height, starch content and total pentosan content among 220 test-cross progenies each, obtained using F**_**3:4**_ **from two bi-parental segregating populations, population A and population B**

Traits	Mean	Range				h ^2^
	Population A					
Grain yield	79.3	73.4 - 85.4	3.33**	8.40**	3.48	0.75
Plant height	118.2	110.9 - 126.6	7.30**	4.03**	2.94	0.92
Starch content	61.6	60.0 - 62.9	0.24**	0.26**	0.14	0.87
Total pentosan content	10.0	9.5 - 10.6	0.03**	0.08**	0.04	0.75
	Population B					
Grain yield	75.6	69.5 - 83.4	3.76**	9.18**	3.72	0.75
Plant height	115.6	104.5 - 127.4	12.62**	4.02**	2.22	0.96
Starch content	61.5	59.8 - 63.4	0.44**	0.24**	0.14	0.93
Total pentosan content	10.3	9.6 - 11.0	0.03**	0.09**	0.04	0.73

refers to the genotypic variance,  represents the interaction variance between genotype and environment, and  denotes the variance of effective error.

**Significantly different from zero with *P* < 0.01.

### Genotypic data analysis

Each of the two times 220 F_3:4_ lines in population A and population B had been genotyped with simple sequence repeat (SSR), single nucleotide polymorphism (SNP) and diversity array technology (DArT) markers [5]. We reapplied quality checks to these marker data, excluding markers with (i) a rate of missing values above 5 % and (ii) allele frequencies smaller than 0.05 or larger than 0.95, and complemented missing genotypes according to a binomial distribution. If not indicated otherwise, only data from DArT markers (394 for population A, 584 for population B, and 1048 for combined populations A and B) were included in analysis. Linkage disequilibrium (LD) was estimated using the squared allele frequency correlations (*r*^2^) [24]. The LD structures in population A, population B, and combined populations A and B are provided in Additional file 1: Figure S1.

### Marker-assisted selection

Marker-assisted selection based on QTL (MAS-QTL) was performed within population B in context with QTL mapping *via* PLABQTL [25] employing composite interval mapping (CIM) by the regression approach [26] in combination with the use of cofactors [27, 28]. We contrasted MAS based on the detected QTL, with MAS based on a random sample of molecular markers. This "neutral marker-assisted selection" (MAS-NEUT) uses markers that were randomly selected according to the number of QTL identified with the respective limit of detection (LOD) score in MAS-QTL for the corresponding trait (first one from each of the seven linkage groups in rye, then randomly chosen additional ones in the case of more than seven QTL). For MAS-QTL, cross-validation was implemented within PLABQTL [25] and accuracies of prediction were calculated as  , where *R*^2^_CV_ denotes the percentage of phenotypic variance the test set explained by identified QTL and *h*^2^ represents heritability [5]. For MAS-NEUT, cross-validation was implemented within population B according to scheme CV_G_ Within-Within as described below.

### Genomic selection

Breeding values were estimated by model, *y* = *μ*1_*N*_ + *Xa* + *e*, where *y* is an *N* × 1 vector of BLUEs estimated across environments; *μ* represents overall mean, 1_*N*_ refers to a vector with the length *N*, *a* is the marker effect, *X* refers to a design matrix for the marker effect, and *e* denotes a residual. By using ridge regression best linear unbiased prediction (RR-BLUP) [29], the estimated marker effects ( ) were estimated based on a mixed model equation, , where  is the transpose of 1_*N*_, *X*^T^ represents the transpose of *X*, *I* is an identity matrix, *λ* represents a penalty parameter, and  denotes the estimated overall mean. The penalty parameter can be calculated as , where *m* is the number of markers and *h*^2^ refers to the heritability of the estimation set [30]. Then the genetic values were predicted as , where  is the estimated marker effect.

### Cross-validation

In all cross-validation approaches, data sets were divided into an estimation set (ES) that was used to estimate marker effects, and a test set (TS), in which the predictive ability (Pearson correlation *r*_MP_) between observed BLUEs and the genotypic values predicted based on the determined marker effects was calculated to provide a measure of the accuracy of prediction [11]. Correlations were either determined as accuracy of prediction *r*_p_ = *r*_MP_ or as standardized accuracy of prediction*r*_g_ = *r*_MP_/*h* calibrated by the square root of heritability [10, 31, 32]. Sampling of estimation and test sets was repeated 5,000 times in each cross-validation scheme.

For cross-validation across genotype (CV_G_), data sets were split into five equally sized subsets (S1 to S5). Four subsets (S1-S4) comprised the ES for estimating marker effects, while the remaining subset (S5) served as TS. Members of the ES and TS were taken either from individual populations (CV_G_ Within-Within) from both populations (CV_G_ Across-Across), or the ES was taken from both populations, population A and B, while the TS was taken from one population (CV_G_ Across-Within). In the case that ES and TS were from individual populations (CV_G_ Within-Within) they could either originate from the same population (CV_G_ Within-Within-Same) or from different populations (CV_G_ Within-Within-Different).

Cross-validation across genotype based on different numbers of environments (CV_G_ Env) was conducted separately within population A and population B (CV_G_ Within-Within-Same). Data for the ES and TS were taken from one to nine randomly permutated location-year combinations. Cross-validation across genotype within location [CV_G(L)_], across genotype and location (CV_G×L_), across genotype within year [CV_G(Y)_] and across genotype and year (CV_G×Y_) was also implemented separately within population A and population B (CV_G_ Within-Within-Same). For cross-validation across genotype and within location [CV_G(L)_] or across genotype and location (CV_G×L_), data for the ES were derived from two randomly selected locations over the two years 2010 and 2011 (e.g. BEK10, BEK11, PET10, and PET11), while the data for the TS were taken either from the same location-year combinations [CV_G(L)_] (e.g. BEK10, BEK11, PET10, and PET11), or from the remaining four location-year combinations (CV_G×L_) (e.g. WAL10, WAL11, PET10, and PET11, but always excluding HOH10). In contrast, for cross-validation across genotype and within year [CV_G(Y)_] or across genotype and year (CV_G×Y_), data for the ES were collected from one year, either 2010 or 2011, at four locations (e.g. BEK10, PET10, WAL10, and WOH10), with data for the TS taken either from the same year [CV_G(Y)_] (e.g. BEK10, PET10, WAL10, and WOH10, but always excluding HOH10) or from the other year (CV_G×Y_) (e.g. BEK11, PET11, WAL11, and WOH11).

## Results

### Field trials and genotyping analysis

Field trials with hybrid rye from test-crosses involving 220 F_3:4_ members and their two parental elite inbred lines of bi-parental segregating population A and population B, respectively, were performed at five locations in Germany and Poland in the years 2010 and 2011 as reported in detail by Miedaner et al. [5]. High quality evaluation data with heritabilities in the range from 0.73 to 0.96 (Table 1) were obtained from nine location-year combinations for grain yield and plant height and from six location-year combinations for starch content and total pentosan content. Populations A and B were derived from crosses Lo115-N × Lo90-N and Lo115-N × Lo117-N, respectively, sharing one common parent, Lo115-N. Thus, their members can be considered as half-sibs. Both populations were characterized by the presence of broad genotypic variance  as well as interaction variance between genotype and environment  for grain yield, plant height, and starch content, and, to a lesser extent, total pentosan content (Table 1). For all traits analyzed, significant (*P* < 0.01) genotypic variance and variation due to genotype × environment interaction effects was obtained in both populations. Consistent with this, genotypic values for the four traits covered broad ranges (Additional file 2: Figure S2). Medians for all traits differed between the two populations, in particular with regard to grain yield, but genotypic variations indicated by the 50%-quartile were quite similar. Except for grain yield, genotypic values of the parents of both populations were rather close to the median. Genotyping based on 1048 molecular markers revealed 394 markers segregating among members of population A and 584 markers segregating among members of population B.

### Standarized accuracy of prediction of marker-assisted selection in comparison to genomic selection

**Figure 1:**
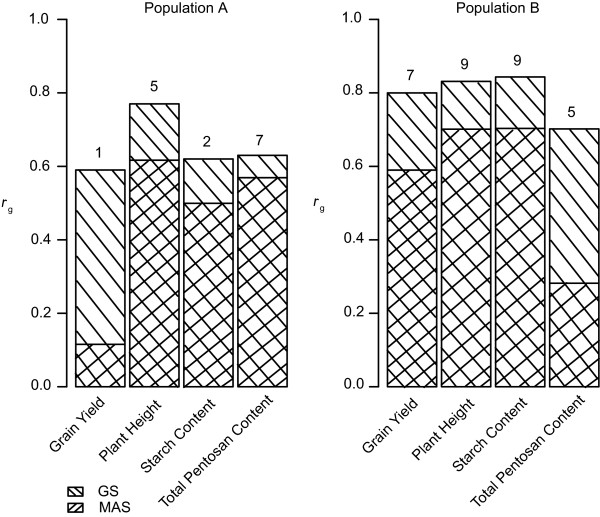
**Cross-validated standardized accuracies of prediction (** ***r***_***g***_ **) for genomic selection compared to marker-assisted selection.** Genomic selection (GS) was based on ridge regression best linear unbiased prediction (RR-BLUP), while marker-assisted selection (MAS) was based on QTL mapped with a limit of detection (LOD) threshold of 3.73 and the detected QTL (numbers above columns). Cross-validation was performed separately within population **A** and population **B** (CV_G_ Within-Within-Same) for traits grain yield, plant height, starch content, and total pentosan content. Accuracies of prediction for MAS were taken from [5] and were based on SSR and DArT markers for population **A** and on SSR and SNP markers for population **B**.

Cross-validated accuracies of prediction based on marker-assisted selection (MAS) for grain yield, plant height, starch content, and total pentosan content in hybrid rye from test-crosses involving the two segregating populations have been reported previously [5]. Performing cross-validated genomic selection (GS) using RR-BLUP [29] based on the very same dataset, we found consistently higher standardized accuracies of prediction in both populations for all four traits that were analyzed (Figure 1). In particular, GS increased the standardized accuracy of prediction from 0.12 with MAS to 0.59 for grain yield in population A and from 0.28 with MAS to 0.70 for total pentosan content in population B. Comparable, albeit less drastic, increases of accuracy were obtained with GS for all other traits in both populations. In order to further explore the potential limitations of MAS, the standardized accuracies of prediction by MAS based on mapped QTL (MAS-QTL) for population B were compared to the results of neutral marker-assisted selection (MAS-NEUT) performed based on randomly selected markers distributed equally across linkage groups over a range of limit of detection (LOD) values, which also generated substantial standardized accuracies of prediction (Additional file 3: Figure S3). Thus, taking into account the consistently lower standardized accuracies of predictions of MAS in combination with the potential over-estimation, all further analysis was based on GS using RR-BLUP.

### Accuracy of prediction of genomic selection within and across populations

**Figure 2:**
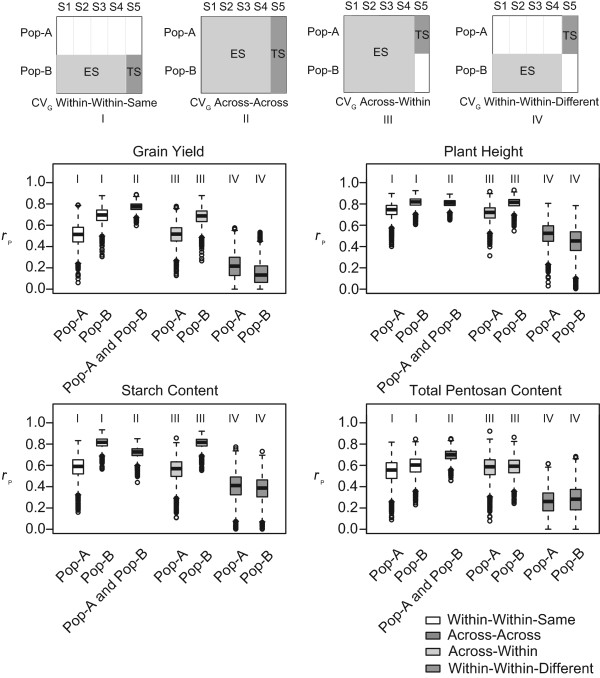
**Cross-validated accuracies of prediction (** ***r***_***p***_ **) for genomic selection using RR-BLUP across genotype (CV**_**G**_ **).** Accuracies of prediction were determined within and across populations. ES in the schemes on top refers to the estimation set, TS represents the test set. In each case, the dataset was divided into five subsets (S1-S5), of which S1 to S4 were assigned to the ES and S5 to the TS. Box-whisker plots of accuracy of prediction *r*_p_ values for traits grain yield, plant height, starch content, and total pentosan content based on data from population **A** (Pop-A) and population **B** (Pop-B) follow below. Cross-validation was performed with estimation and test sets derived from one population, either Pop-A or Pop-B (CV_G_ Within-Within-Same; white; category I), estimation and test sets derived from both populations, Pop-A and Pop-B (CV_G_ Across-Across; dark grey; category II), the estimation set derived from both populations, Pop-A and Pop-B, and the test set from one population, either Pop-A or Pop-B (CV_G_ Across-Within; light grey; category III), or estimation set derived from one population, either Pop-A or Pop-B, and the test set from one population, either Pop-B or Pop-A (CV_G_ Within-Within-Different; intermediate grey; category IV).

Taking advantage of the unique design of the two bi-parental segregating populations sharing one parental inbred line in common, we applied CV_G_ with different estimation set-test set combinations in order to study the dependency of the accuracy of prediction of GS on the relatedness of the included material (Figure 2). Separate cross-validation across genotype among sibs only within each population (CV_G_ Within-Within-Same; Figure 2, category I) showed consistently lower accuracies of prediction for population A than population B for all four traits. For example, the median accuracy of prediction *r*_*p*_ was approximately 0.51 for population A and 0.70 for population B for grain yield and approximately 0.75 for population A and 0.82 for population B for plant height, respectively. Cross-validation across genotype among combined sibs and half-sibs with estimation and test sets taken from both populations (CV_G_ Across-Across; Figure 2, category II) generated a slight increase of the accuracies of prediction in the cases of grain yield and total pentosan content, but accuracies of prediction for the other two traits were approximately intermediate between the accuracies of prediction for sibs within populations A and B, respectively, in CV_G_ Within-Within-Same. When the estimation set was extended across the two populations to include sibs and half-sibs, but the test set was restricted to sibs from one population only (CV_G_ Across-Within; Figure 2, category III), the accuracies of prediction were essentially the same as when estimation set and test set were from sibs from the same population in CV_G_ Within-Within-Same. Finally, when the estimation set was taken from within one population and the test set from the other population (CV_G_ Within-Within-Other; Figure 2, category IV) for prediction among half-sibs only, accuracies of prediction were substantially lower than in the CV_G_ Within-Within-Same scenario among sibs for all traits analyzed.

### Accuracy of prediction of genomic selection across genotypes based on different numbers of location-year combinations

**Figure 3:**
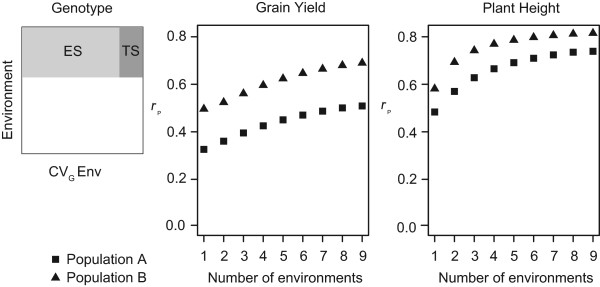
**Cross-validated accuracies of prediction for genomic selection using increasing environment numbers (CV**_**G**_ **Env).** Calculation was done with RR-BLUP across genotype. ES in the scheme at the left refers to the estimation set, TS represents the test set. At the right, the impact of increasing the number of environments used for cross-validation on the accuracy of prediction *r*_p_ for grain yield and plant height in population **A** (squares) and population **B** (triangles).

In order to judge the contribution of the extension of field trials to the accuracy of prediction of genomic selection, cross-validation across genotype was performed separately within population A and population B for grain yield and plant height based on increasing numbers of environments, that is, location-year combinations (CV_G_ Env; Figure 3). Accuracies of prediction were consistently lower for population A in comparison to population B. They increased continuously with the number of included environments, with the gain per added environment being lower for grain yield and higher for plant height.

### Accuracy of prediction of genomic selection within or across locations and years

**Figure 4:**
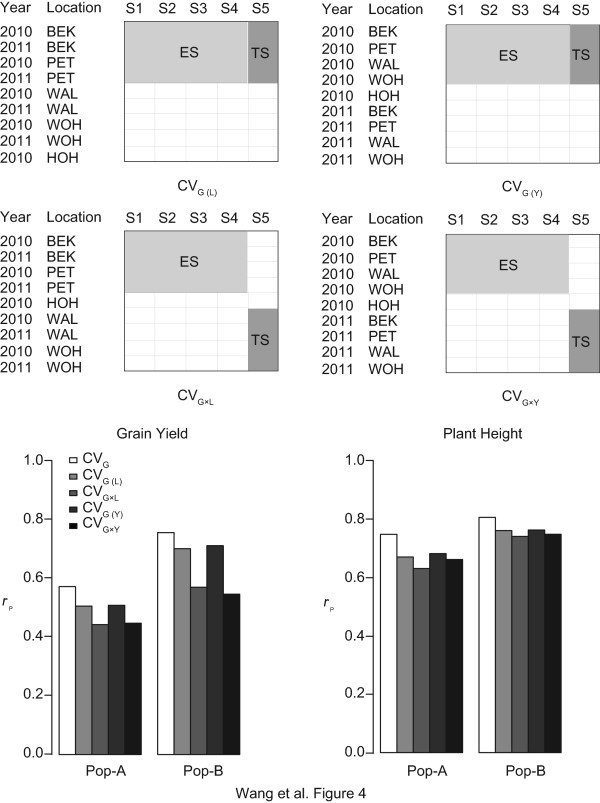
**Cross-validated accuracies of prediction for genomic selection with regard to location and year.** Cross-validation was performed using RR-BLUP across genotype within location [CV_G (L)_] or across genotype and location (CV_G×L_), and across genotype within year [CV_G (Y)_] or across genotype and year (CV_G×Y_). ES in the scheme on top refers to the estimation set, TS represents the test set. In each case, the dataset was divided into five subsets (S1-S5), of which S1 to S4 were assigned to the ES and S5 to the TS. The mean accuracy of prediction *r*_p_ values for genomic selection based on data from population A (Pop-**A**) and population **B** (Pop-B) for traits grain yield and plant height are shown below. The mean *r*_p_ across genotype based on the eight location-year combinations for which data from two years could be included (BEK10, BEK11, PET10, PET11, WAL10, WAL11, WOH10, WOH11; excluding HOH10) serves as reference (CV_G_; white). The mean *r*_p_ across genotype within location [CV_G (L)_] or across genotype and location (CV_G×L_) was determined by selecting data from two locations in the years 2010 and 2011 (e.g. BEK10, BEK11, PET10, PET11) for estimation, the mean *r*_p_ across genotype within year [CV_G (Y)_], or across genotype and year (CV_G×Y_) from estimation sets based on data from four locations in one year (e.g. BEK10, PET10, WAL10, WOH10). Test sets were derived either from the same location-year combinations as the estimation sets [CV_G (L)_; light grey; CV_G (Y)_; dark grey] or from the location-year combinations not used for estimation (CV_G×L_; intermediate grey; CV_G×Y_; black).

The availability of data sets from five locations over two years for grain yield and plant height allowed us to estimate the effects of limiting the number of locations or number of years on accuracies of prediction, which is pivotal for the optimal allocation of resources in field trials. Analysis was done separately within either population A or population B based on estimation sets from four location-year combinations. Test sets were taken from the same four location-year combinations [CV_G(L)_ and CV_G (Y)_; Figure 4], or from the remaining four available location-year combinations excluding HOH10 (CV_G×L_ and CV_G×Y_; Figure 4). The accuracy of prediction of GS across genotype within location [CV_G(L)_] or across genotype and location (CV_G×L_) was determined by selecting data from two locations in the years 2010 and 2011 for estimation, the accuracy of prediction of GS across genotype within year (CV_G(Y)_) or across genotype and year (CV_G×Y_) was derived from estimation based on data from four locations in 2010 or 2011. Accuracies of prediction in CV_G(L)_ were approximately the same as in CV_G(Y),_ and accuracies of prediction in CV_G×L_ were nearly the same as in CV_G×Y_ in both populations for each of the analyzed traits. When estimation and test sets were collected from the different location-year combinations (CV_G×L_ and CV_G×Y_), accuracies of prediction were consistently lower in comparison to the situation with both estimation and test sets form the same location-year combinations [CV_G(L)_ and CV_G(Y)_].

## Discussion

The superior standardized accuracy of prediction of GS in comparison to MAS for the complex traits grain yield, plant height, starch content, and total pentosan content in rye (Figure 1) is in line with observations for diverse traits in other crops [33, 34]. The limitations of MAS in comparison to GS have been discussed in detail previously [7–9, 35]. In the context of our study, the analysis of MAS-NEUT based on randomly selected markers revealed a substantial contribution to the standardized accuracy of prediction that is not dependent on informative markers linked to QTL (Additional file 3: Figure S3). With MAS-NEUT representing a kind of special case of genomic selection, this might be due to the relatedness of genotypes in the respective population [18, 36–38].

Focusing on genomic selection *via* RR-BLUP, we explored accuracies of prediction within and across bi-parental populations as well as across field trial locations and years. The accuracies of prediction for population-specific test-sets were rather similar, no matter whether estimation was done within (CV_G_ Within-Within-Same) or across populations (CV_G_ Across-Within) (Figure 2). A reason for this could have been the close relationship of population A and population B, being half-sibs sharing one parental line (Lo115-N). However, accuracies of prediction were consistently higher for test-sets from population B. This cannot be explained by variation in the quality of field trials, as heritabilities seen with hybrids derived from population A and population B were similar for all four traits considered (Table 1). A possible explanation for the higher accuracy would be the presence of higher genetic diversity in population B in comparison to population A. This view is supported by the higher genetic variance  in population B for three of the four traits in study as well as the higher number of segregating molecular markers (584 versus 394). Consistently, population A was generated by crossing two superior test-cross lines, Lo115-N and Lo90-N, while population B was obtained by crossing one superior line, Lo115-N with a lower performing line, Lo117-N [5]. A similar positive effect of higher genetic variation on the accuracy of prediction in genomic selection has been reported by Zhao et al. [39] and Riedelsheimer et al. [40]. However, also the higher number of polymorphic markers in population B *per se* might contribute to higher accuracy of prediction [41]. Actually, the rather low number of markers used in analysis might present a general limitation of the accuracy of genomic selection in our current study. This limitation could be overcome by taking advantage of recently developed SNP arrays for rye [42]. When genomic selection was done solely across populations (CV_G_ Across-Across), either a slight decrease or a slight increase of the accuracy of prediction was observed depending on the trait. This could be due to the difference among the two populations. For grain yield and total pentosan content, accuracies of prediction were higher in CV_G_ (Across-Across) than in CV_G_ (Within-Within-Same) and CV_G_ (Across-Within). This could be caused by a larger genetic diversity among populations in comparison to the genetic diversity within each population. In contrast, for plant height, the genetic variation among two populations was not quite large, and for starch content the genetic variation among two populations was rather small, leading to accuracies of prediction in CV_G_ (Across-Across) between the accuracies of prediction of CV_G_ (Within-Within-Same) and CV_G_ (Across-Within). When genomic selection was attempted from one population to the other (CV_G_ Within-Within-Different), accuracies of prediction were consistently lower than in all other approaches, readily revealing the limits of predicting among half-sib populations in hybrid rye. This is consistent with the in general rather pessimistic prognosis for GS-based prediction for material distantly related to the plants evaluated in field trials in diverse crops [40, 43, 44].

With the continuous reduction of genotyping costs over time, phenotypic evaluation in field trials has now become the more cost-intensive action in the calibration of MAS or GS in plant breeding programs [45, 46]. As there is consistent pressure to reduce costs, reduction of field trial expenses is a tempting option. In addition, the established phenotypic selection schemes usually allow only one year of field testing for the vast majority of selection candidates. However, cross-validation testing of the impact of field trial size on the accuracy of prediction of genomic selection (CV_G_ Env) as indicated by *r*_p_ in our study revealed a clear dependence on the number of location-year combinations for hybrid rye (Figure 3). The grade of this dependence varied for the analyzed traits in hybrid rye. In the case of plant height, increases of accuracy were only marginal beyond the inclusion of data from five environments, while in the case of grain yield, the accuracy was still substantially increasing when all available data from nine environments were included, underlining the need for testing over a sufficient number of location-year combinations for optimal calibration. An explanation for the difference between the two traits might lie in the lower heritability seen for grain yield in comparison to plant height (Table 1).

In order to separately check the specific impacts of the number of field trial locations and the number of test years on the prediction accuracy, we performed cross-validated genomic selection across genotype within location [CV_G(L)_], or across genotype and location (CV_G×L_) using data from two out of four locations for estimation, and across genotype within year [CV_G (Y)_], or across genotype and year (CV_G×Y_) using data from one out of two years (Figure 4). The accuracies of prediction were consistently higher in CV_G(L)_ and CV_G(Y)_ with test sets from the same subset of locations or the same year, respectively, than in CV_G×L_ and CV_G×Y_, with the test set taken from the locations or the year not included in the estimation set. Thus, the accuracy of prediction was limited by genotype and location and genotype and year interactions. A similar limitation from location to location prediction accuracy has been reported for MAS in hybrid maize [11]. According to Ly et al. [44], the "overestimation" of the accuracy of prediction resulting from taking estimation and test sets from the same environments can be determined based on the magnitude of genotype and environment interaction effect (G × E interaction) . It varies substantially for different traits. Based on the  and  values from Table 1, the ratio of G × E interaction for grain yield was 0.72 and 0.71 in population A and population B, respectively, compared to a ratio of G × E interaction for plant height of 0.36 and 0.24 in population A and population B in our study. The larger ratio of G × E interaction of grain yield in comparison to plant height explains thus the larger decrease of the accuracy of prediction of GS for grain yield than plant height from one set of evaluation locations to other locations or one testing year to another [CV_G×L_ compared to CV_G(L)_ and CV_G×Y_ compared to CV_G (Y)_; Figure 4]. In this context, it has to be considered that testing in only one year limits the accuracy of prediction, indication that in hybrid rye testing for more than one year is of importance to the optimal calibration of genomic selection. This is consistent with observations on limitations set by available data from evaluation locations and testing years for maize made by Kleinknecht et al. [47].

## Conclusions

In hybrid rye, genomic selection is superior to marker-assisted selection which generates lower accuracies of prediction which are potentially overestimated. However, high accuracies of prediction are achieved by genomic selection only for candidates closely related to the plant material evaluated in field trials, resulting in a rather pessimistic prognosis for distantly related material. As both, the number of evaluation locations and the number of testing years contribute equally to accuracy field trials for the calibration of genomic selection should be performed in more than one year at several locations.

## Electronic supplementary material

Additional file 1: Figure S1: Linkage disequilibrium (LD) structure for diversity array technology (DArT) markers. Data was based on 394 and 584 segregating markers within population A and population B, respectively, and 1048 markers across both populations. (PDF 107 KB)

Additional file 2: Figure S2: Genotypic values for grain yield, plant height, starch and total pentosan content. Hybrid rye derived from test-crosses of two segregating bi-parental populations was analysed. Data were collected for test-cross progenies from two times 220 F_3:4_ lines and their respective parents across nine (for grain yield and plant height) or six (for starch content and total pentosan content) environments, respectively. P1 and P2 refer to the parental lines of population A (Pop-A, Lo115-N x Lo90-N; white), P1 and P3 the parental lines of population B (Pop-B, Lo115-N x Lo117-N; grey). (PDF 116 KB)

Additional file 3: Figure S3: Cross-validated standardized accuracies of prediction (*r*_*g*_) for QTL-based versus random marker-assisted selection. QTL-based marker-assisted selection (MAS-QTL) was performed in comparison to marker-assisted selection performed based on randomly sampled neutral markers (MAS-NEUT). Cross-validation was performed within population B (CV_G_ Within-Within-Same) for traits grain yield, plant height, starch content, and total pentosan content. QTL mapping based on estimation set data was performed using different limit of detection (LOD) thresholds (numbers below columns), resulting in ranges of median numbers of detected QTL (numbers in the boxes above columns). Analysis was based on 900 DArT markers as described in [5]. (PDF 172 KB)

## Abbreviations

BEK: Beckedorf
BLUEs: Best linear unbiased estimates
CMS: Cytoplasmatic-genic male sterility
CV: Cross-validation
CV_G_: Cross-validation across genotype scheme
CV_G_ Across-Across: Cross-validation across genotype scheme in which members of the ES and TS are taken from both populations
CV_G_ Across-Within: Cross-validation across genotype scheme in which ES are taken from both populations, while the TS are taken from one population
CV_G_ Env: Cross-validation across genotype scheme based on different numbers of environments
CV_G_ Within-Within: Cross-validation across genotype scheme in which members of the ES and TS are taken from either individual population
CV_G_ Within-Within-Different: CV_G_ Within-Within scheme in which ET and TS are collected from the different population
CV_G_ Within-Within-Same: CV_G_ Within-Within scheme in which ET and TS originate from the same population
CV_G (L)_: Cross-validation across genotype within location scheme in which data for the ES is derived from two randomly selected locations over the two years 2010 and 2011, while the data for the TS is taken either from the same location-year combinations
CV_G×L_: Cross-validation across genotype and location scheme in which data for the ES is derived from two randomly selected locations over the two years 2010 and 2011, while the data for the TS is from the remaining four location-year combinations
CV_G (Y)_: Cross-validation across genotype within year scheme in which data for the ES was collected from one year, either 2010 or 2011 with data for the TS taken either from the same year
CV_G×Y_: Cross-validation across genotype and year scheme in which data for the ES was collected from one year, either 2010 or 2011 with data for the TS taken either from the other year
DArT: Diversity array technology
ES: Estimation set
G × E interaction: Genotype and environment interaction
GS: Genomic selection
HOH: Hohenheim
LD: Linkage disequilibrium
MAS: Marker-assisted selection
MAS-NEUT: Neutral marker-assisted selection
MAS-QTL: Marker-assisted selection based on QTL
PET: Petkus
QTL: Quantitative trait loci
RR-BLUP: Ridge regression best linear unbiased prediction
SNP: Single nucleotide polymorphism
SSR: Simple sequence repeat
TS: Test set
WAL: Walewice
WHO: Wohlde.
