# Supplementary figures and images for: The accuracy of prediction of genomic selection in elite hybrid rye populations surpasses the accuracy of marker-assisted selection and is equally augmented by multiple field evaluation locations and test years
Source: BMC Genomics. 2014 Jul 4;15(1):556. doi: 10.1186/1471-2164-15-556 (PMC4101178; doi:10.1186/1471-2164-15-556)

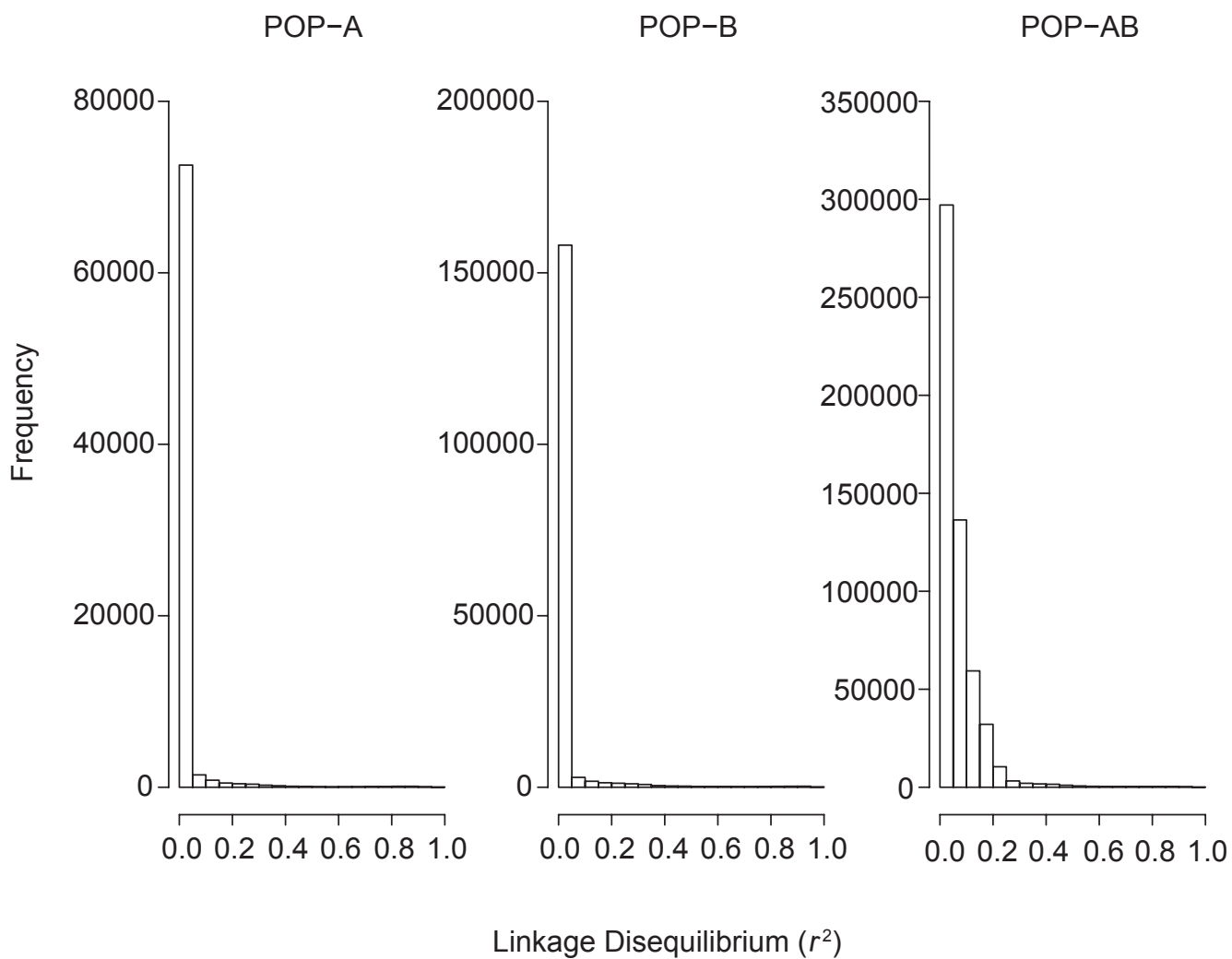

Supplement: Supplementary file 1 — Additional file 1: Figure S1: Linkage disequilibrium (LD) structure for diversity array technology (DArT) markers. Data was based on 394 and 584 segregating markers within population A and population B, respectively, and 1048 markers across both populations. (PDF 107 KB) [file 12864_2014_6247_MOESM1_ESM.pdf]

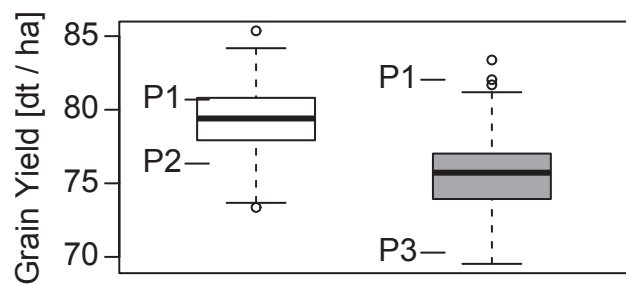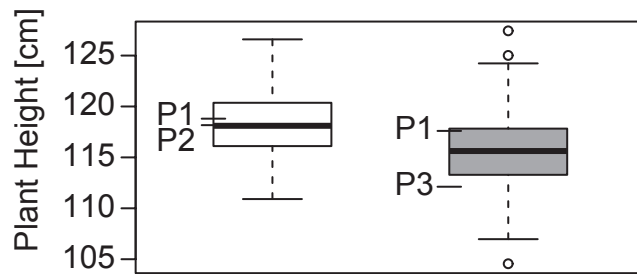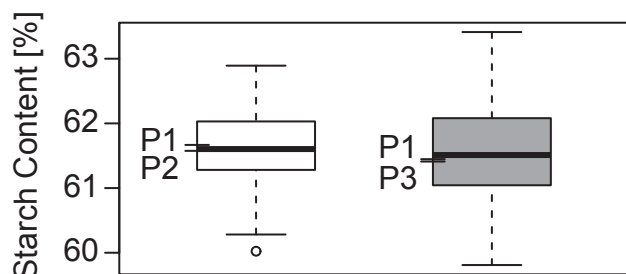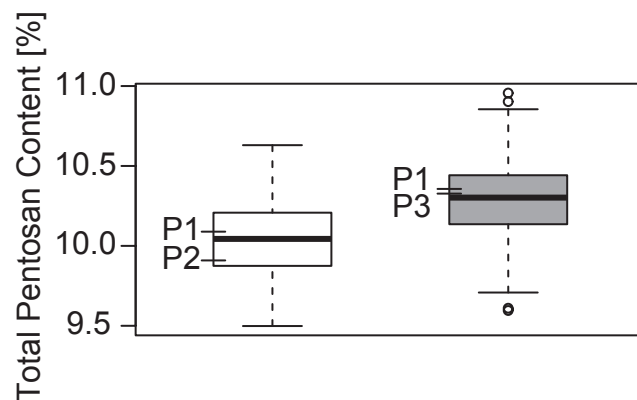

□ Pop-A  
■ Pop-B

Supplement: Supplementary file 2 — Additional file 2: Figure S2: Genotypic values for grain yield, plant height, starch and total pentosan content. Hybrid rye derived from test-crosses of two segregating bi-parental populations was analysed. Data were collected for test-cross progenies from two times 220 F3:4 lines and their respective parents across nine (for grain yield and plant height) or six (for starch content and total pentosan content) environments, respectively. P1 and P2 refer to the parental lines of population A (Pop-A, Lo115-N x Lo90-N; white), P1 and P3 the parental lines of population B (Pop-B, Lo115-N x Lo117-N; grey). (PDF 116 KB) [file 12864_2014_6247_MOESM2_ESM.pdf]

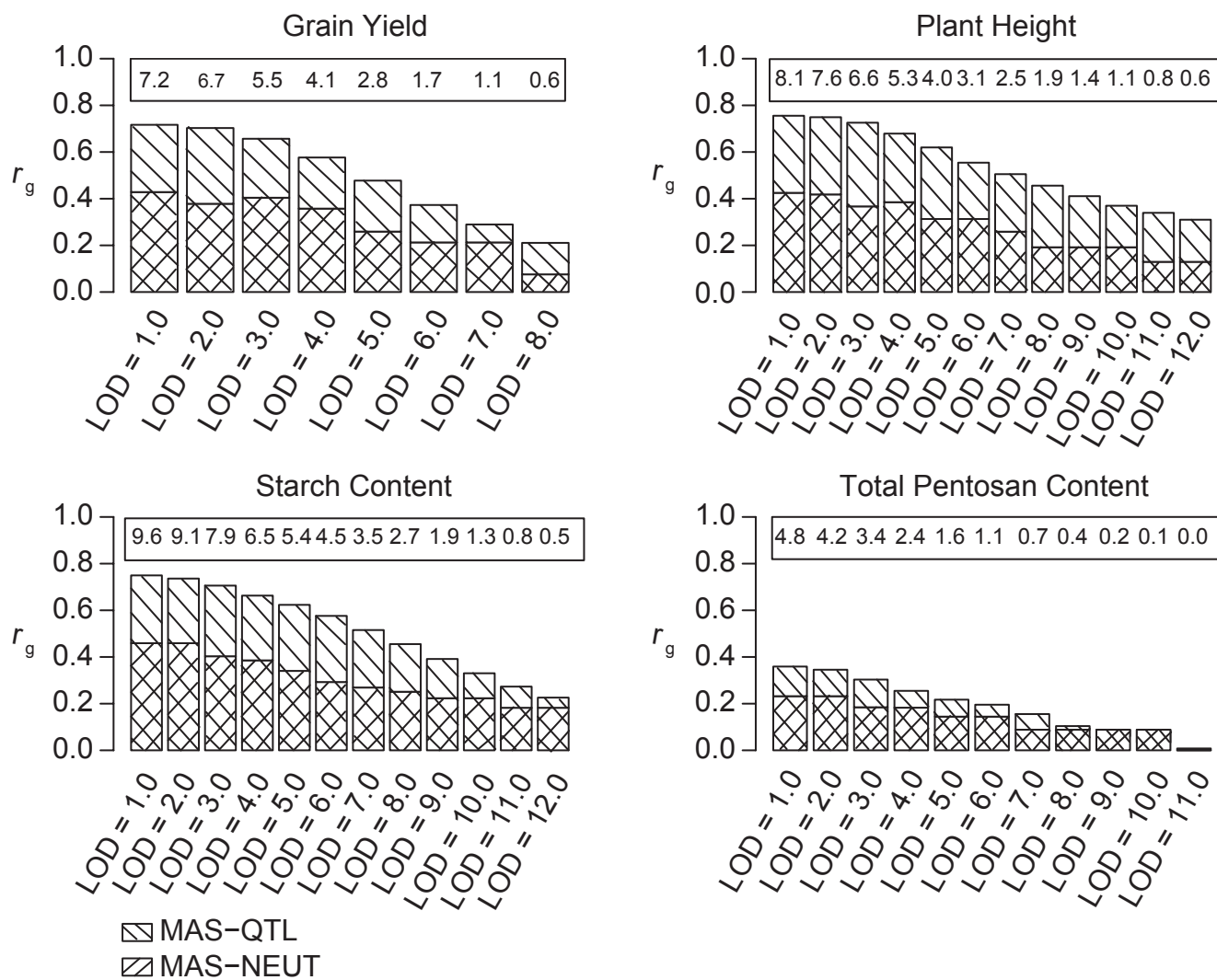

Wang et al. Figure S3

Supplement: Supplementary file 3 — Additional file 3: Figure S3: Cross-validated standardized accuracies of prediction (r g) for QTL-based versus random marker-assisted selection. QTL-based marker-assisted selection (MAS-QTL) was performed in comparison to marker-assisted selection performed based on randomly sampled neutral markers (MAS-NEUT). Cross-validation was performed within population B (CVG Within-Within-Same) for traits grain yield, plant height, starch content, and total pentosan content. QTL mapping based on estimation set data was performed using different limit of detection (LOD) thresholds (numbers below columns), resulting in ranges of median numbers of detected QTL (numbers in the boxes above columns). Analysis was based on 900 DArT markers as described in [5]. (PDF 172 KB) [file 12864_2014_6247_MOESM3_ESM.pdf]
